# Sepsis-associated hospitalisations and antimicrobial use in Hong Kong

**DOI:** 10.1017/S0950268821002193

**Published:** 2021-10-11

**Authors:** Weixin Zhang, Peng Wu, Celine S. L. Chui, Wey Wen Lim, Benjamin J. Cowling

**Affiliations:** 1 WHO Collaborating Centre for Infectious Disease Epidemiology and Control, School of Public Health, Li Ka Shing Faculty of Medicine, The University of Hong Kong, Hong Kong Special Administrative Region, China; 2 Laboratory of Data Discovery for Health Limited, Hong Kong Science Park, New Territories, Hong Kong Special Administrative Region, China; 3 Department of Pharmacology and Pharmacy, Centre for Safe Medication Practice and Research, Li Ka Shing Faculty of Medicine, The University of Hong Kong, Hong Kong Special Administrative Region, China; 4 Department of Paediatrics and Adolescent Medicine, Li Ka Shing Faculty of Medicine, The University of Hong Kong, Hong Kong Special Administrative Region, China; 5 Department of Social Work and Social Administration, Faculty of Social Sciences, The University of Hong Kong, Hong Kong Special Administrative Region, China

**Keywords:** Antimicrobial, hospitalisation, prescription, sepsis

## Abstract

Our study was conducted to assess the sepsis-associated hospitalisations and antimicrobials prescribed for sepsis inpatients in Hong Kong. Demographic, diagnostic and antimicrobial prescription data were analysed for patients admitted to public hospitals with a diagnosis of septicaemia from 2000 to 2015. A total of 223 250 sepsis hospitalisations were recorded in Hong Kong from 2000 to 2015 during which the hospitalisation rate increased by 85.6%. The majority of the sepsis hospitalisations occurred in adults ≥65 years and children aged 0–4 years. Adults with a secondary diagnosis of sepsis were often admitted with a primary diagnosis of urological conditions or pneumonia; whereas diabetes mellitus was the most common secondary diagnosis among those with primary sepsis. Paediatric sepsis patients aged 0–4 years were often diagnosed with disorders relating to short gestation and low birthweight. Antimicrobial prescriptions increased by 51.1% and 34.4% for primary and secondary sepsis patients, respectively. *β*-Lactam and *β*-lactamase inhibitor combinations were the most used antibiotics whereas the usage of carbapenems increased more than 10 times over the study period. A substantial burden of hospitalisations was attributable to sepsis in Hong Kong, particularly in the extremes of age. Broad-spectrum and last-resort antibiotics had been increasingly dispensed for sepsis inpatients.

## Introduction

Sepsis is the systemic inflammatory response to infection, often being referred to interchangeably with septicaemia in clinical practice. Although the analysis of septicaemia rates has been limited by the clinical relevance of the term, septicaemia is explicitly coded in the *International Classification of Diseases, Ninth Revision* (ICD-9) [1]. Sepsis is a major contributor to morbidity and mortality in hospital worldwide [2]. It was estimated that 48.9 million sepsis cases and 11.0 million sepsis-related deaths occurred in 2017 worldwide [3], with variable estimates in developed countries and a lack of data from developing countries.

People at the highest risk of developing sepsis include the very young and the very old, particularly those with comorbidities, an impaired immune system or indwelling catheters [4]. Respiratory infections were the most common source infection of sepsis in China and the USA, followed by infection of the abdomen and genitourinary tracts [5, 6]. *Staphylococcus aureus*, *Pseudomonas* species and *Escherichia coli* were common pathogens associated with sepsis [4]. As a critical condition primarily treated in a hospital, sepsis involves a substantial amount of antibiotic use and is therefore the main target of antimicrobial stewardship programmes [7]. Early and broad-spectrum empirical antibiotic therapy is essential for the treatment of sepsis [8]. The occurrence and prognosis of sepsis can be affected by the antibiotic resistance profile of the causative pathogens [9].

Sepsis has been one of the leading causes of death in Hong Kong in the last decade [10]. We therefore conducted a study to examine the patterns of hospitalisations and antimicrobial prescriptions associated with sepsis in Hong Kong from 2000 to 2015.

## Methods

### Data sources

Around 80% of bed-days (BDs) in Hong Kong between 2000 and 2015 were in public hospitals, with the remainder in a smaller number of private hospitals [11]. Hospital admission data of patients who were admitted to a public hospital with a discharge diagnosis of septicaemia (ICD-9: 038) from 2000 to 2015 were acquired from the Hospital Authority of Hong Kong which managed all local public hospitals [11]. Previous studies indicated that the ICD-9 code (038) could identify over 90% of the patients diagnosed with sepsis [12, 13]. We extracted information including sex, age group, the year and month of admission, length of hospital stay and individual discharge diagnoses including the primary diagnosis and up to 14 additional secondary diagnoses. All diagnoses were coded by the ICD-9 whereas only the initial three digits of the code were collected to protect the privacy of the patients. In-hospital antimicrobial prescription records were also obtained for individual septicaemia patients, including the dispensing order of prescribed antimicrobials during admission, time of dispensing, drug name, frequency, route of administration and dispensing duration in days (only available from 2003 and onwards). We also collected monthly numbers of all-cause hospital admissions and admissions with antimicrobials prescribed by age group in all public hospitals from 2000 to 2015. The age- and sex-specific annual population size during the study period was obtained from the Census and Statistics Department of Hong Kong [14]. We defined age groups as 0–4, 5–19, 20–44, 45–64, 65–84 and ≥85 years. Microbiological data were not available for patients in this study.

We classified the hospital admissions into primary sepsis and secondary sepsis based on the patients' diagnoses. We classified patients as having primary sepsis if they were discharged from the hospital with septicaemia as the principal diagnosis, indicating sepsis being the main reason contributing to the treatment and investigation during the admission. Patients were classified as having secondary sepsis if they were discharged with septicaemia as one of the secondary diagnoses which were likely to develop during hospital stay or as a complication of care received in the hospital. Other diagnoses were extracted to characterise the clinical profile of all the patients with sepsis based on methods given in Supplementary Table S1.

Antimicrobials prescribed for the patients were classified into 11 groups according to the mechanism of action and indications of the antimicrobials, including antibiotics by type, antivirals, antifungals and anti-parasitics.

The study was approved by the Institutional Review Board of the University of Hong Kong/Hospital Authority Hong Kong West Cluster (HKU/HA HKW IRB) (ref. UW 16-369).

### Statistical analysis

We estimated the annual hospitalisation rates of overall, primary and secondary sepsis by age group and sex from 2000 to 2015 with the mid-year population as the denominator and the annual numbers of sepsis admissions as the numerator. The crude age- and sex-specific hospitalisation rates were standardised based on the age and sex distributions of the Hong Kong population in 2015. An upgraded electronic system for medical management has been used by public hospitals in Hong Kong since 2009. Therefore, our analysis of the hospitalisation rates was conducted separately for the two periods: 2000–2008 and 2009–2015. The negative binomial regression model was used to investigate the variations in annual sepsis hospitalisations in different age, sex and sepsis groups. We examined the temporal pattern of sepsis hospitalisation and explored the effect of changes in coding practices in 2009 and later on sepsis hospitalisation. The annual proportions of all-cause hospitalisations due to sepsis were also estimated in comparison with other primary causes of admission.

We characterised the profiles of patients with primary and secondary sepsis who might have experienced different clinical courses by examining the demographics of the patients and all co-existing diagnoses. The distributions of hospital stay and antimicrobial prescriptions were also estimated by patients' characteristics. The World Health Organization (WHO) recommends the defined daily dose (DDD) as an indicator for drug utilisation [15]; however, it only reflects drug use for defined major indications among adults, and the drug consumption measured by DDD could be biased when the actual dose dispensed to patients varied significantly from the WHO-recommended doses. The drugs dispensed in Hong Kong public hospitals were coded according to the British National Formulary in which a WHO-recommended dose may not be available for all the listed drugs [16]. Therefore, we used days of therapy (DOT) per 1000 hospital BDs to measure antimicrobial use for patients hospitalised with sepsis, as previously published [17]. When a patient was prescribed multiple antimicrobials in a day, the number of DOT derived from that specific day of treatment would be equal to the number of antimicrobials received on the day. All analyses were conducted using R 3.5.2 (R Foundation for Statistical Computing, Vienna, Austria).

## Results

From 2000 to 2015, 223 250 admissions to public hospitals in Hong Kong were recorded from 165 420 individual patients with a diagnosis of sepsis, among which half (50.1%) of the admissions had a primary diagnosis of sepsis. About 0.6–1.6% and 0.3–0.8% of the inpatients were discharged with sepsis as a primary or secondary diagnosis in years 2000–2008 and 2009–2015, respectively (Supplementary Fig. S1). Among all patients with primary sepsis, 40.1% and 16.0% did not have any recorded secondary diagnoses during these two periods, respectively. The average number of diagnoses per sepsis admission increased from 3.4 in 2000–2008 to 5.1 in 2009–2015.

The age- and sex-standardised overall sepsis hospitalisation rate had increased by 85.6% from 159.0 per 100 000 person-years in 2000 to 296.1 per 100 000 person-years in 2015, peaking at 361.0 per 100 000 person-years in 2010 (Supplementary Fig. S2). The standardised hospitalisation rate of primary sepsis gradually increased from 95.7 per 100 000 person-years in 2000 to 145.8 per 100 000 person-years in 2015, with an annual increase of 1.0% (95% confidence interval (CI) −0.5 to 2.4). The standardised hospitalisation rate of primary sepsis in 2009–2015 is 16.5% (95% CI 1.7–33.4) higher as compared with that in 2000–2008. The standardised hospitalisation rate of secondary sepsis, however, showed an abrupt rise in 2009 onwards and is, on average, 86.8% (95% CI 49.5–133.0, *P* < 0.001) higher in 2009–2015 than that during 2000–2008, with an annual increase of 2.5% (95% CI 0–5.1) during 2000 and 2015. Over the same time period, the age-standardised all-cause hospitalisation rate in the public hospitals only slightly increased by 1.4% (21.0% in 2000, 22.4% in 2015), with the average annual hospitalisation rate in 2009–2015 17.4% (95% CI 6.3–28.9) higher than that in earlier years.

**Fig. 1.**
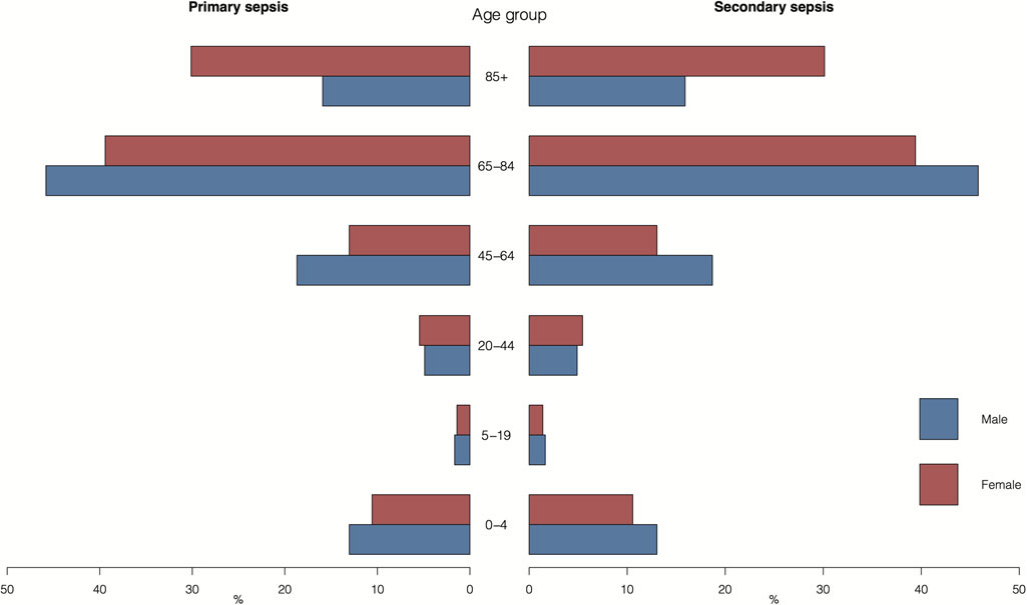
Age and sex distribution of primary and secondary sepsis inpatients.

**Fig. 2.**
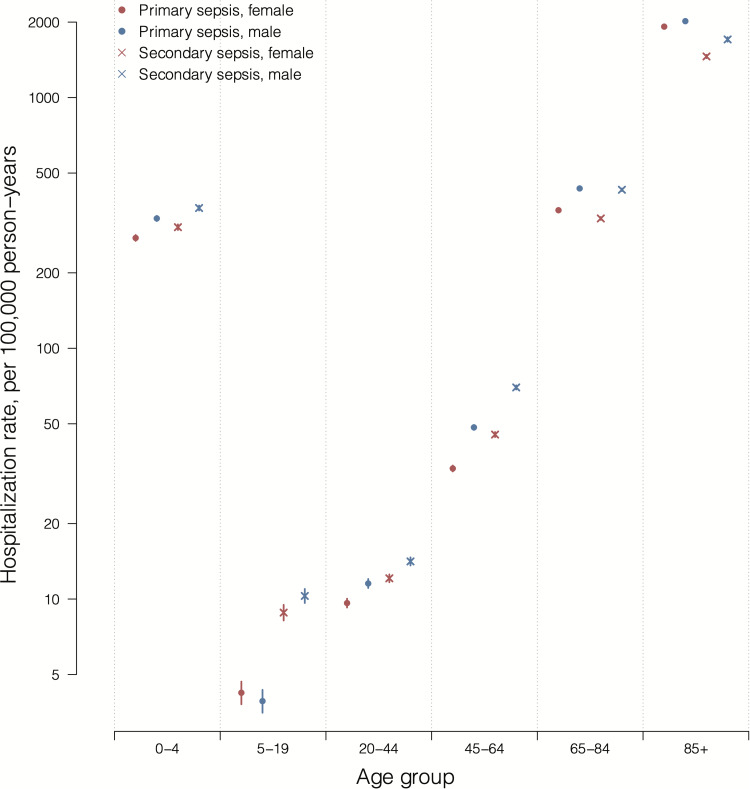
Annual average hospitalisation rates of patients with primary and secondary sepsis by age group and sex on log scale, 2000–2015.

Overall, 69.4% of the sepsis hospitalisations were reported in adults aged ≥65 years, with the smallest proportion (4.7%) in individuals 5–19 years of age (Fig. 1). The annual hospitalisation rates of primary sepsis were the highest in adults ≥85 years and in the youngest children 0–4 years old (Fig. 2). The lowest rate was reported in young adults 5–19 years of age with an average annual rate of 4.1 admissions per 100 000 person-years. The secondary sepsis hospitalisation rates increased in most age groups especially from 2009 onwards whereas the rate in children aged 0–4 years remained relatively stable over the study period (Supplementary Fig. S3).

The overall average annual hospitalisation rates of sepsis (primary and secondary) were generally higher in men compared to women particularly in those aged 0–4, 45–64 and 65–84 years, and higher proportions of hospitalisations were in males compared to females in these age groups except for the aged ≥85 years (Fig. 2). The median length of hospital stay was similar between male and female patients with primary sepsis, whereas female patients with secondary sepsis showed slightly shorter median hospital stay than their male counterparts (Supplementary Table S2). Compared to patients with secondary sepsis, patients with primary sepsis were older, more likely to be women, and showing a shorter median hospital BDs.

We examined recorded diagnoses for all the sepsis patients. Other disorders of urethra and urinary tract (ICD-9: 599) were the most commonly reported diagnosis for both primary and secondary sepsis hospitalisations, and pneumonia was the second most frequently recorded diagnosis among adult patients with secondary sepsis (Table 1). Among patients with primary sepsis aged 64 years and above, diabetes mellitus and cerebrovascular diseases were the leading co-diagnoses (15.5% and 23.6% of patients ≥65 years were diagnosed with the two conditions, respectively). About one-third (34.5%) of the sepsis patients at age of 5–19 years had at least one diagnosis related to neoplasms, and myeloid leucaemia (ICD-9: 205) was the most frequently recorded diagnosis in them. The majority (62.7%) of the sepsis patients at 0–4 years had conditions originating in the perinatal period or disorders relating to short gestation, and low birthweight (ICD-9: 765) was the leading diagnosis.

**Table 1.** Top 10 primary diagnoses of patients with secondary sepsis, 2000–2008 and 2009–2015

2000–2008		2009–2015	
Diagnoses	Frequency (%)	Diagnoses	Frequency (%)
599 Other disorders of urethra and urinary tract	5102 (14.42)	599 Other disorders of urethra and urinary tract	7978 (10.49)
765 Disorders relating to short gestation and low birthweight	2349 (6.64)	486 Pneumonia organism unspecified	6618 (8.7)
486 Pneumonia organism unspecified	1678 (4.74)	585 Chronic kidney disease (CKD)	2316 (3.04)
585 Chronic kidney disease (CKD)	753 (2.13)	576 Other disorders of biliary tract	2012 (2.65)
780 General symptoms	723 (2.04)	780 General symptoms	1960 (2.58)
250 Diabetes mellitus	714 (2.02)	250 Diabetes mellitus	1649 (2.17)
428 Heart failure	710 (2.01)	427 Cardiac dysrhythmias	1551 (2.04)
434 Occlusion of cerebral arteries	637 (1.8)	482 Other bacterial pneumonia	1507 (1.98)
276 Disorders of fluid electrolyte and acid-base balance	598 (1.69)	401 Essential hypertension	1460 (1.92)
519 Other diseases of respiratory system	588 (1.66)	574 Cholelithiasis	1363 (1.79)

**Fig. 3.**
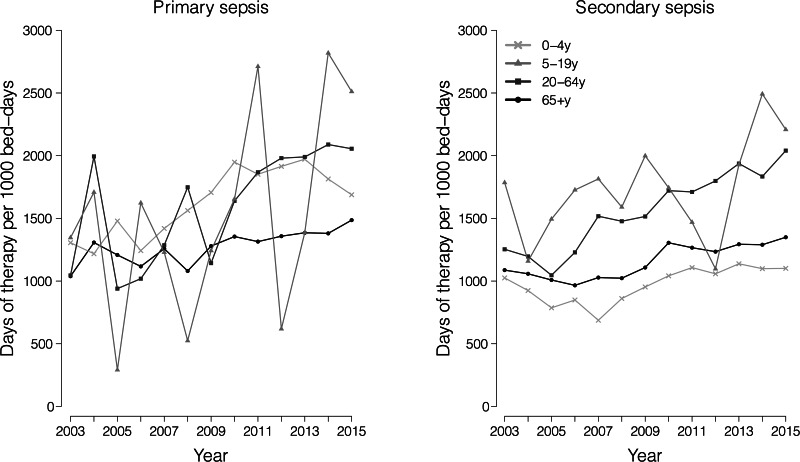
Days of antimicrobial therapy per 1000 BDs for patients with primary and secondary sepsis by age group, 2003–2015.

In total, 2 623 347 antimicrobial prescriptions were dispensed for the sepsis inpatients from 2000 through 2015. Among the dispensed antimicrobials, 93.9% were antibiotics, accounting for 7.4% of all antibiotic prescriptions recorded in the public hospitals during the study period. There were 882 720 prescriptions for 111 683 patients with primary sepsis, and most of the prescriptions (67.6%) were for patients ≥65 years old. Although the median hospital BDs were largely stable across the study years, the overall antimicrobial prescriptions measured by DOT per 1000 BDs increased 51.1% and 34.4% for patients with primary and secondary sepsis, respectively. The annual average DOTs were 1057.0 days per 1000 BDs in 2003, and 1597.7 days per 1000 BDs in 2015 in patients of primary sepsis, and 1120.3 and 1501.6 days per 1000 BDs for secondary sepsis hospitalisations in 2003 and 2015, respectively. The estimated DOT per 1000 BDs did not vary substantially across age groups although the youngest group with primary sepsis had consistently longer DOT per 1000 BDs compared to the elderly aged 65 and above over the study period. In contrast, children at age of 0–4 years with secondary sepsis largely had the shortest DOT per 1000 BDs among all age groups (Fig. 3).

*β*-Lactam and *β*-lactamase inhibitor combinations were the most frequently prescribed antibiotics for both primary and secondary sepsis over the study period, and the prescriptions doubled from 2003 to 2015 (Table 2). The most commonly used antimicrobials following prescription of *β*-lactam and *β*-lactamase inhibitor combinations were third–fifth generation cephalosporins, first–second generation cephalosporins and quinolones for patients with either primary or secondary sepsis. Carbapenems had been prescribed for sepsis patients from all the age groups since 2003 particularly those aged 20 years and above. The usage of carbapenems rose up from 9.6 DOT per 1000 BDs and 24.8 DOT per 1000 BDs in 2003 for primary and secondary sepsis patients to almost 12 times and 5 times higher in 2015, respectively (Table 2). Prescriptions of first–second generation cephalosporins had decreased more than 50% in both types of sepsis patients in 2003–2015. The use of the third–fifth generation cephalosporins in primary sepsis patients doubled (increased 109%) in 2015 compared to year 2003 whereas remained largely similar for secondary sepsis patients over the same time period. Tetracyclines and glycopeptides were relatively less often prescribed, but prescriptions of both were more than doubled among primary sepsis patients in 2015 in comparison with year 2003.

**Table 2.** Days of therapy (DOT) with antimicrobials per 1000 bed-days (BDs) for patients with primary and secondary sepsis by class of antimicrobial, 2003 and 2015

Antimicrobial class	Primary sepsis			Secondary sepsis		
	Year 2003	Year 2015	Percentage change	Year 2003	Year 2015	Percentage change
Carbapenems	9.6	114.1	1089	24.8	119.2	381
Non-antibiotics^a^	37.1	129.1	248	107.5	236.5	120
*β*-Lactam and *β*-lactamase inhibitors combinations	313.5	712.3	127	195.7	467.0	139
Glycopeptides	21.9	49.5	126	80.0	73.5	−8
Third–fifth generation cephalosporins	51.6	107.9	109	83.3	86.7	4
Tetracyclines	10.5	20.1	91	3.0	14.8	393
Quinolones	96.3	113.8	18	76.6	113.5	48
Other antibiotics^b^	117.0	116.8	−0.2	160.5	211.6	32
Aminoglycosides	67.2	66.4	−1	137.6	55.0	−60
Penicillins	139.9	95.0	−32	124.3	65.5	−47
First–second generation cephalosporins	140.7	54.7	−61	94.7	41.3	−56
Macrolides	51.6	18.1	−65	32.3	16.9	−48

a Including antivirals, anti-fungus and anti-parasitics.

b Including anti-tuberculosis agents, aztreonam, chloramphenicol, colistin, daptomycin, fosfomycin, fusidic acid, lincosamides, linezolid, metronidazole, nitrofurantoin, rifaximin, sulphonamides, trimethoprim and vancomycin.

## Discussion

Our analysis identified a considerable burden of hospitalisations associated with patients discharged with a diagnosis of septicemia in Hong Kong, with a stable increase in primary sepsis hospitalisation rates over the study period and an abrupt rise in secondary sepsis hospitalisation rates likely due to the changes of coding practice in the hospitals. Other developed countries also reported high levels of incidence of sepsis [2, 18]. A recent study in the USA reported the primary sepsis hospitalisation rates in 2001–2014 comparable to what we estimated in this study, but with a considerable change over time [6]. Temporal increases in sepsis hospitalisations have also been reported in other countries, although case ascertainment might vary across the studies [19, 20].

The Hospital Authority of Hong Kong has developed the Clinical Management System (CMS) since the 1990s to support electronic health records [21]. The CMS reached Phase III by operating a full web-based and service-oriented healthcare management system in 2009. Our analysis demonstrated that hospitalisation rates of primary sepsis appeared to be relatively stable over the years. In contrast, secondary sepsis hospitalisations had a drastic increase in more recent years in 2009 and onwards, with a similar increase in the average number of diagnoses per primary hospitalisation. The 86.8% increase in secondary sepsis hospitalisations that occurred in 2009–2015 was potentially attributable to the upgrade of the medical record management system, implying that a substantial proportion of secondary sepsis patients might have been underreported before 2009 in Hong Kong.

Sepsis hospitalisations occurred at higher rates in the extremes of age [4]. The increasing sepsis rates in patients at age of 65 years and above in Hong Kong in more recent years were largely due to the increase in the hospitalisation rate of secondary sepsis. Chronic diseases were often co-diagnosed in the hospitalised sepsis patients as either the primary or a secondary diagnosis [22], or were potential risk factors of sepsis [23]. The incidence of urinary tract infections (UTIs) was relatively high among older people who were admitted to hospital for treatment of chronic diseases [24]. Despite a generally low incidence in progressing to sepsis among UTI patients, approximately 25% of the adult sepsis patients were due to UTIs (urosepsis) [25]. UTIs and respiratory infections, as the most common causes of sepsis [1], often led to prescriptions of antibiotics in the community and primary care settings [26].

Our study showed that primary sepsis patients, on average, received more than one antibiotic prescription per day during the entire hospital stay. It is particularly concerning that the youngest patients received the highest numbers of antibiotic prescriptions throughout the admission [27]. Early-life exposure to antibiotics could alter the microbiome of infants and negatively contribute to the health status in adulthood [28]. Although the infant mortality rate in Hong Kong has been the lowest in the world [29], the gradually elevating hospitalisation burden and the extensive antibiotic use related to neonatal and paediatric sepsis and their potential impact may need further studies.

It is also concerning that more and broader antibiotics had been prescribed to sepsis patients in Hong Kong particularly carbapenems. Globally, consumption of carbapenems in retails and hospital pharmacies was generally low but on the rise [30]. The Hospital Authority of Hong Kong implemented an active surveillance on carbapenem-resistant *Enterobacteriaceae* (CRE) in all public hospitals to screen for carriers in newly admitted high-risk patients [31]. The relatively low carriage of CRE in the screened patients (<1%) during 2010–2011 had increased considerably to 8% in 2015, and the carriage was associated with the previous use of cephalosporins or carbapenems [31]. Antimicrobials including fluoroquinolones, third-generation cephalosporins and carbapenems were also shown to be associated with the occurrence of CRE infections in Europe and Japan [32, 33]. Therefore, the direct and indirect impacts of carbapenem use on the risk of infection with CRE in hospitalised patients should be further investigated.

Our study is subject to several limitations. First, we might have missed sepsis patients admitted to private hospitals. However, we believe this would not change the main findings of our study because almost all critical emergencies and about 80% of all hospital admissions in Hong Kong were managed by the public hospitals under the Hospital Authority [11]. Second, the data did not allow us to analyse the diagnoses beyond the first three digits of the ICD-9 codes, which limited the ability to examine more detailed diagnoses and to illustrate possible sources of infection or exact comorbidities. Third, microbiological test results were not available for the patients in this study, preventing us from examining the aetiology of sepsis and investigating its association with antibiotic prescription. However, a limited added value was expected even if microbiological data were available because no causative pathogens were identified in around 70% of inpatients with sepsis who were admitted to a local tertiary hospital (personal communication), and the percentage of sepsis patients with positive blood culture was reported to be as low as 17% in the USA [34]. Finally, we were unable to determine antibiotics prescribed for other purposes and the length of hospital stay before the onset of sepsis, particularly secondary sepsis occured after hospital admission, and therefore might overestimate antibiotic use attributable to sepsis. The overestimation was, however, believed to be limited considering that a much larger amount of antibiotics is often prescribed for patients with sepsis than those with other infections/procedures.

In conclusion, sepsis was associated with a substantial burden of hospitalisation in Hong Kong from 2000 to 2015, especially among patients at the extremes of age. Pneumonia and urinary tract infections were likely to be the main sources of infection leading to sepsis whereas attention should be paid to older patients with underlying conditions to avoid occurrence of sepsis. The increasing use of broader spectrum antibiotics in sepsis inpatients warrants further investigations on the resistance profile of pathogenic bacteria and the health impact of a large amount of antibiotic exposure on these patients.

## Data Availability

The dataset used for this analysis will not be publicly available as the authors do not have the ownership of the data.

## Supplementary material

For supplementary material accompanying this paper visit http://dx.doi.org/10.1017/S0950268821002193. click here to view supplementary material
