# Supplementary material for: Sepsis-associated hospitalisations and antimicrobial use in Hong Kong
Source: Epidemiol Infect. 2021 Oct 11;149:e231. doi: 10.1017/S0950268821002193 (PMC8569835; doi:10.1017/S0950268821002193)

Supplementary material

Table of contents Page

Supplementary Table S1. ICD-9 diagnostic codes for comorbidities of sepsis patients **2**

Supplementary Table S2. Characteristics of sepsis inpatients admitted into public hospitals in Hong Kong, 2000-2015, by primary and secondary sepsis **3-4**

Supplementary Figure S1. Monthly proportions of hospital admissions public hospitals in Hong Kong by primary diagnosis, 2000-2015 **5**

Supplementary Figure S2. Age- and sex-standardized annual hospitalization rates with 95% confidence intervals of overall, primary and secondary sepsis in Hong Kong from 2000 through 2015 **6**

Supplementary Figure S3. Annual hospitalization rates of patients with primary and secondary sepsis by age group and sex, 2000-2015 **7**

Supplementary Table S1. ICD-9 diagnostic codes for comorbidities of sepsis patients

Co-diagnoses	ICD-9 diagnostic codes
Neoplasms	140-239 (Neoplasms), V66 (Convalescence and palliative care)
Diabetes mellitus	250 (Diabetes mellitus)
Hypertensive disease	401-405 (hypertensive disease)
Cardiovascular & cerebrovascular diseases	410-414 (Ischemic Heart Disease), 427 (Cardiac dysrhythmias), 428 (Heart failure), 434 (Occlusion of cerebral arteries), 438 (Late effects of cerebrovascular disease)
Pneumonia & influenza	480-488 (Pneumonia and influenza)
Disorders of gallbladder and biliary tract	574 (Cholelithiasis), 575 (Other disorders of gallbladder), 576 (Other disorders of biliary tract)
Chronic kidney disease & renal failure	584 (Acute kidney failure), 585 (Chronic kidney disease), 586 (Renal failure, unspecified)
Urinary disorders	599 (Other disorders of urethra and urinary tract)
Disorders originating in the prenatal period	760-779 (Certain conditions originating in the perinatal period), V30 (Single liveborn)
General symptoms	780 (General symptoms)

Supplementary Table S2. Characteristics of sepsis inpatients admitted into public hospitals in Hong Kong, 2000-2015, by primary and secondary sepsis

	Age group	Primary sepsis		Secondary sepsis	
		**Male**	**Female**	**Male**	**Female**
Number of admission[a] (Proportion, %)	0-4	6742 (12.5)	5221 (9.0)	7420 (13.0)	5763 (10.6)
	5-19	357 (0.7)	364 (0.6)	938 (1.6)	760 (1.4)
	20-44	2275 (4.2)	2368 (4.1)	2786 (4.9)	2973 (5.5)
	45-64	7370 (13.7)	5207 (9.0)	10,640 (18.7)	7110 (13.0)
	65-84	26,392 (49.9)	23,176 (40.0)	26,078 (45.8)	21,489 (39.4)
	85+	10,712 (19.9)	21,607 (37.3)	9056 (15.9)	16,431 (30.1)
	Overall[a]	100	100	100	100
Annual average hospitalization rate, per 100,000 person-years (95% CI)	0-4	329.6 (321.7-337.5)	275.5 (268.1-283.1)	362.7 (354.5-371.1)	304.1 (296.3-312.1)
	5-19	3.9 (3.5-4.3)	4.2 (3.8-4.7)	10.3 (9.6-11.0)	8.8 (8.2-9.5)
	20-44	11.5 (11.1-12.0)	9.6 (9.3-10.0)	14.1 (13.6-14.7)	12.1 (11.7-12.5)
	45-64	48.4 (47.3-49.5)	33.2 (32.3-34.1)	69.8 (68.5-71.2)	45.3 (44.2-46.4)
	65-84	434.1 (428.8-439.3)	355.4 (350.8-360.0)	428.9 (423.7-434.1)	329.5 (325.1-334.0)
	85+	2017.3 (1979.3-2055.9)	1918.4 (1892.9-1944.2)	1705.5 (1670.5-1741.0)	1458.8 (1436.6-1481.3)
	Overall[b]	125.1 (124.2-126.1)	119.5 (118.7-120.4)	125.1 (124.2-126.1)	105.6 (104.8-106.4)
Days of hospital stay, median (IQR)	0-4	7.0 (5.0-8.0)	7.0 (5.0-8.0)	14.0 (7.0-40.0)	15.0 (7.0-42.0)
	5-19	6.0 (3.0-11.0)	6.0 (3.0-11.0)	14.0 (6.0-36.0)	12.0 (5.0-34.0)
	20-44	5.0 (3.0-9.0)	5.0 (3.0-8.0)	9.0 (4.0-22.0)	7.0 (4.0-17.0)
	45-64	6.0 (3.0-10.0)	6.0 (3.0-10.0)	11.0 (5.0-23.0)	9.0 (5.0-20.0)

	65-84	6.0 (3.0-11.0)	6.0 (4.0-11.0)	10.0 (5.0-20.0)	9.0 (5.0-18.0)
	85+	6.0 (3.0-11.0)	6.0 (3.0-10.0)	8.0 (5.0-17.0)	8.0 (4.0-15.0)
	Overall	6.0 (4.0-10.0)	6.0 (4.0-10.0)	10.0 (5.0-22.0)	9.0 (5.0-19.0)
No. of discharge diagnoses, mean (SD)	0-4	2.7 (1.7)	2.5 (1.7)	6.4 (3.9)	6.4 (3.9)
	5-19	2.8 (2.5)	2.6 (2.1)	5.6 (3.8)	5.3 (3.5)
	20-44	2.5 (2.0)	2.6 (2.0)	5.0 (3.1)	4.6 (2.9)
	45-64	3.4 (2.7)	3.4 (2.5)	5.7 (3.2)	5.4 (3.0)
	65-84	3.5 (2.6)	3.5 (2.6)	5.6 (3.1)	5.4 (3.0)
	85+	3.6 (2.7)	3.4 (2.5)	5.5 (3.1)	5.1 (2.8)
	Overall	3.4 (2.6)	3.3 (2.5)	5.7 (3.3)	5.3 (3.1)
No. of antibiotic classes prescribed, median (IQR)	0-4	2 (1-2)	2 (1-2)	2 (1-3)	2 (1-3)
	5-19	2 (1-3)	2 (1-2)	2 (1-3)	2 (1-4)
	20-64	2 (1-3)	2 (1-3)	2 (1-4)	2 (1-3)
	65+	1 (1-2)	1 (1-2)	2 (1-3)	2 (1-3)
	Overall	2 (1-2)	1 (1-2)	2 (1-3)	2 (1-3)
Days of antibiotic therapy (DOT)/1000 bed-days	0-4	1722.2	1594.4	976.3	916.4
	5-19	1238.1	1043.5	1602.3	1763.0
	20-64	1435.8	1747.1	1655.8	1679.8
	65+	1356.7	1224.1	1268.6	1130.0
	Overall	1404.7	1314.1	1316.4	1211.9

[a]11 admissions unknown of age were excluded.

[b]Standardized to the age and sex distribution of 2015 population.

Supplementary Figure S1. Monthly proportions of hospital admissions public hospitals in Hong Kong by primary diagnosis, 2000-2015.

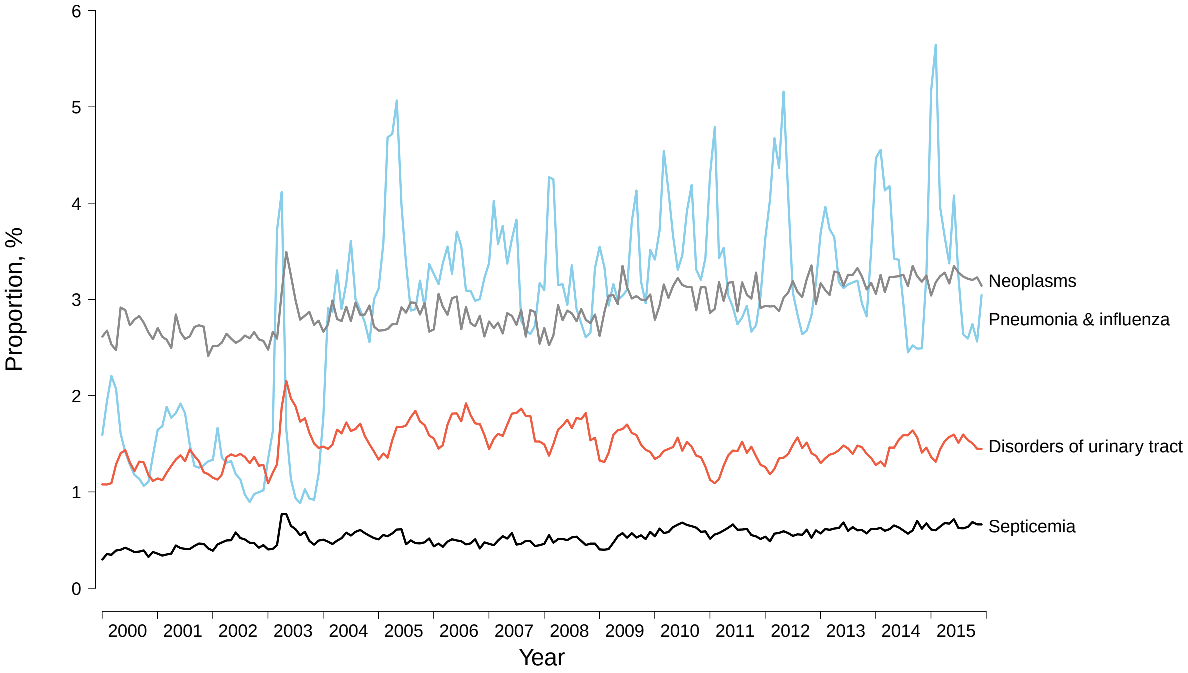

Supplementary Figure S2. Age- and sex-standardized annual hospitalization rates with 95% confidence intervals of overall, primary and secondary sepsis in Hong Kong from 2000 through 2015.

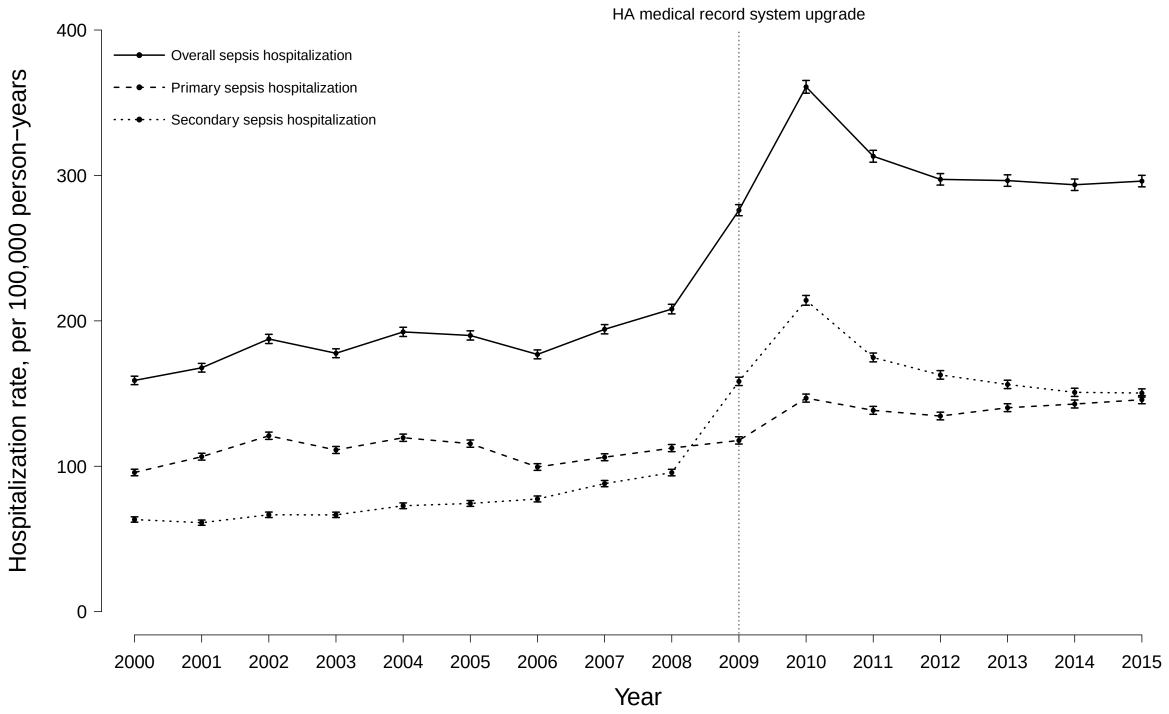

Supplementary Figure S3. Annual hospitalization rates of patients with primary and secondary sepsis by age group and sex, 2000-2015

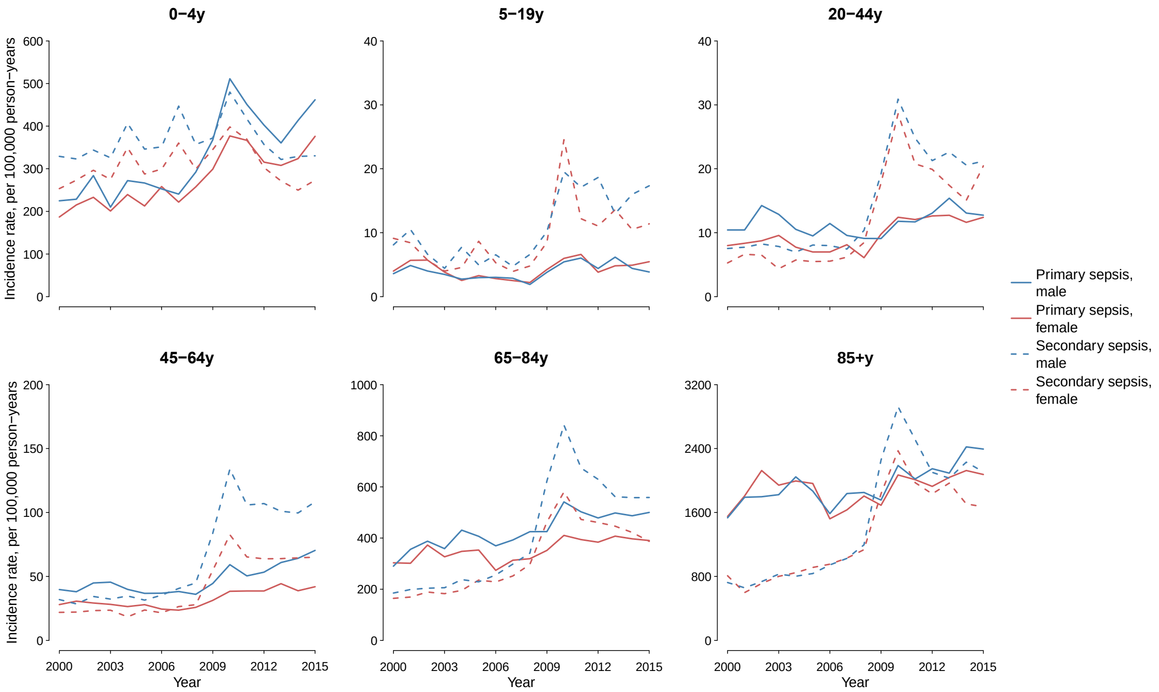

Supplement: Supplementary file 1 [file S0950268821002193sup001.pdf]
